# IntiCom-DB: A Manually Curated Database of Inter-Tissue Communication Molecules and Their Communication Routes

**DOI:** 10.3390/biology12060833

**Published:** 2023-06-08

**Authors:** Changxian Xiong, Yiran Zhou, Yu Han, Jingkun Yi, Huai Pang, Ruimao Zheng, Yuan Zhou

**Affiliations:** 1 Department of Biomedical Informatics, Center for Noncoding RNA Medicine, School of Basic Medical Sciences, Peking University, Beijing 100191, China; xcx@bjmu.edu.cn (C.X.); yrzhou@bjmu.edu.cn (Y.Z.); sx_hanyu@bjmu.edu.cn (Y.H.); 1810305301@pku.edu.cn (J.Y.); 2211110088@bjmu.edu.cn (H.P.); 2 State Key Laboratory of Vascular Homeostasis and Remodeling, Peking University, Beijing 100191, China; 3 Department of Anatomy, Histology and Embryology, School of Basic Medical Sciences, Peking University, Beijing 100191, China; rmzheng@pku.edu.cn

**Keywords:** inter-tissue communication, database, communication route, secretory protein, exosome

## Abstract

**Simple Summary:**

Molecules mediating long-distance inter-tissue communication from source to target tissues is essential to coordinate physiological functions and maintain health. A well-known example is insulin, which originates from the pancreas and targets multiple tissues, such as the liver and muscle, in order to control glucose metabolism and to keep the blood glucose level stable. However, knowledge about inter-tissue communication molecules is not always well-organized as in the cited insulin case, and there is currently no specialized database to extensively collect known inter-tissue communication molecules and their communication routes from source tissues to target tissues. In this paper, through a manual literature curation of nearly 190,000 publications, we established a database called IntiCom-DB, which contains 1408 inter-tissue communication relationships supported by experimental evidence. In IntiCom-DB, users can conveniently browse or search for inter-tissue communication molecules and their source tissues, target tissues, functions, and other related information. This database will enhance our understanding of human health and disease biology, and it will facilitate the development of treatments for complex diseases.

**Abstract:**

Inter-tissue communication (ITC) is critical for maintaining the physiological functions of multiple tissues and is closely related to the onset and development of various complex diseases. Nevertheless, there is no well-organized data resource for known ITC molecules with explicit ITC routes from source tissues to target tissues. To address this issue, in this work, we manually reviewed nearly 190,000 publications and identified 1408 experimentally supported ITC entries in which the ITC molecules, their communication routes, and their functional annotations were included. To facilitate our work, these curated ITC entries were incorporated into a user-friendly database named IntiCom-DB. This database also enables visualization of the expression abundances of ITC proteins and their interaction partners. Finally, bioinformatics analyses on these data revealed common biological characteristics of the ITC molecules. For example, tissue specificity scores of ITC molecules at the protein level are often higher than those at the mRNA level in the target tissues. Moreover, the ITC molecules and their interaction partners are more abundant in both the source tissues and the target tissues. IntiCom-DB is freely available as an online database. As the first comprehensive database of ITC molecules with explicit ITC routes to the best of our knowledge, we hope that IntiCom-DB will benefit future ITC-related studies.

## 1. Introduction

In order to maintain physiological homeostasis, different tissues and organs dynamically interact with each other via the long-distance inter-tissue communication (ITC) to globally coordinate their functions and actions [1,2,3,4,5]. A major strategy for ITC is coordinating different tissues via various ITC molecules, including secretory proteins, RNAs, metabolites, and other factors, such as exosomes, that deliver important effectors between physically distant tissues [2,4].

Among classical ITC molecules, secretory proteins are of great biological significance [3,4,5,6,7]. In a study involving 32 human tissues, Uhlén et al. found that secreted proteins accounted for about 10–20% of the coding genome [8]. Following the discovery of insulin, an increasing number of secreted proteins have been identified as inter-tissue signaling molecules and described to regulate nearly all aspects of the physiological activities in mammals [2,6,7,9,10]. Dedicated secretory factor groups such as adipokines [11], myokines [12], and hepatokines [13] are used to summarize the ITC proteins from major source tissues such as adipose tissue, muscle, and liver, respectively. For instance, leptin, an adipokine released by adipose tissue, can regulate the neuroendocrine tissues via the leptin receptor during starvation [14,15]. Similarly, irisin, a myokine produced by skeletal muscle, can drive the browning of white adipose tissue and thermogenesis during exercise [16]. In addition, LCAT, a hepatokine secreted by the liver, is important to the reversal of the bone-to-liver cholesterol transport in hepatic osteodystrophy (HOD) mice [17].

Recent evidence has further extended the scope of ITC molecules beyond secretory proteins. For example, circulating RNAs, especially circulating miRNAs, have recently gained increasing attention as novel ITC factors [3,18,19]. For example, adipose tissue-derived miR-27a can regulate insulin resistance in skeletal muscle [20]. Similarly, heart-derived miR-1 can cause hippocampal microtubule damage and attenuate hippocampal synaptic vesicle exocytosis [21,22]. Various kinds of metabolites, such as lipids, amino acids, ketone bodies, and bile acids also act as signaling molecules for ITC by activating specific membrane receptors [3,4,23,24,25]. The “Cori cycle”, in which lactate molecules produced by skeletal muscle is converted to glucose by the liver and converted back to lactate in the muscle, is the first described example of metabolite-mediated ITC [4,24,26]. Since then, many metabolites have been elucidated to play important roles in ITC. For instance, bile acid salts released by liver will stimulate the expression of FGF15 in the small intestine [27]. In addition, upon cold exposure, free fatty acids produced by white adipose tissue (WAT) can promote hepatic acylcarnitine production, which are, in turn, utilized by brown adipose tissue (BAT) for thermogenesis [28].

Some ITC molecules are protected by extracellular vesicles such as exosomes in order to achieve stable long-range communications. Exosomes are 50–100 nm extracellular vesicles (EVs) surrounded by a phospholipid bilayer, which are released from multivesicular bodies (MVBs) via exocytosis [29]. They can deliver large amounts of biomacromolecules and prevent their cargos from being degraded and inactivated by enzymes in peripheral circulation [4,30]. Indeed, exosomes have been shown to play important roles in ITC by transferring proteins, lipids, RNAs, and other biomolecules to adjacent or to remote cells and tissues [25,30,31]. For example, BAT-derived miR-99b can be delivered by exosomes to the liver to repress the expression of hepatic FGF21 [18], and the exosomes produced by cardiac adipose-derived stem cells contain BDNF, IGF-1, NGF, and GDNF, which can stimulate the proliferation of Schwan cells and promote axonal growth [32].

Compared to the functional importance of ITC in physiological conditions, many diseases, especially metabolic diseases, are extensively regulated by ITC signals [4,25,33]. Briefly, ITC is closely related to the pathological mechanisms of complex diseases, including but not limited to cancers [34,35,36], obesity [37,38,39], diabetes [40,41], and cardiovascular disease [30,42]. Nevertheless, exploration and functional investigations of ITC molecules often require costly and time-consuming experiments, since multiple tissues are involved in. Moreover, and even worse, these experiments are often from different research fields and are related to various pathways. As a result, there is no systematic or comprehensive survey of known ITC molecules and their ITC routes (from the source tissues to the target tissues), at least to the best of our knowledge. 

Therefore, it is urgent and essential to establish a dedicated database for various types of ITC molecules and their explicit ITC routes. To this end, we manually curated the ITC molecules with experiment-supported evidence that have been reported in literature and built a user-friendly ITC molecule database named IntiCom-DB (http://rnanut.net/inticomdb, accessed on 31 May 2023). IntiCom-DB provides an interface by which users can easily browse and query detailed information for all ITC entries.

## 2. Materials and Methods

### 2.1. Literature Data Curation and Annotation

**Figure 1:**
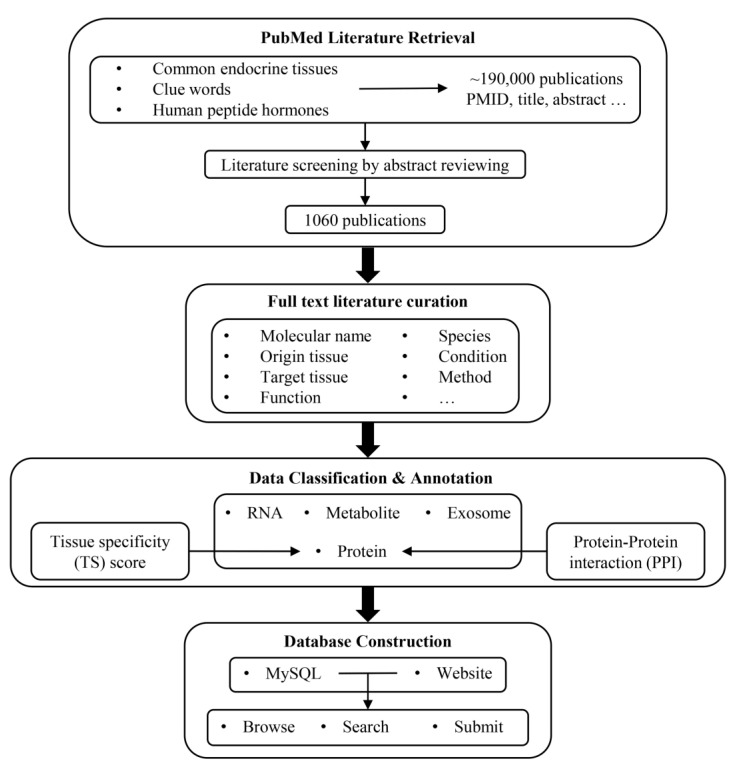
Workflow for constructing IntiCom-DB.

The pipeline for the development of IntiCom-DB is outlined in Figure 1. We first employed two strategies to retrieve ITC-related literature from the PubMed database. The first strategy was to retrieve literature by using single tissues or combining two tissues as keywords, such as “Adipose”, “Liver AND Pituitary”, “Kidney AND Muscle”, etc. The second strategy was to combine words implying inter-tissue interaction relationships, such as “Axis”, “Communication”, “Crosstalk”, and words implying molecules, such as “Protein”, “Peptide”, “Metabolite” and “Exosome” as the keyword combinations for retrieval. The two strategies generated a total of 120 keywords or keyword combinations for retrieval. Through the initial screening by these keywords, a total of 188,728 publications and related information were obtained, including article title, abstract, keywords, etc. The keywords used for retrieval and the number of publications obtained by each group of keywords are shown in Supplementary Table S1. In addition, in order to avoid missing important classical ITC molecules, we retrieved 225 human peptide hormone-related references collected in the HORDB, http://hordb.cpu-bioinfor.org/ (accessed on 9 Jan 2023) human peptide hormone database and screened articles discussing their roles in ITC [43].

Next, we further screened the literature through a manual review of article titles, abstracts, and keywords in order to collect as much information as possible about ITC molecules and more accurate communication pathways. There were two principles for the literature screening: (1) the article simultaneously described the source tissue and target tissue of the ITC molecule, and (2) the ITC molecule described in the article was experimentally verified rather than predicted. To accelerate the literature screening and the extraction of ITC-related information, we used in-house Perl and Linux shell scripts to map and tag gene names, metabolite names, tissue names, and disease names in the literature titles and the abstracts to the corresponding standard professional terms, and then we highlighted them. In addition, words implying interaction relationships were also highlighted in order to provide references and clues for manual review, such as “Axis”, “Communication”, “Crosstalk”, etc. 

Finally, we obtained 1060 works describing ITC molecules and their communication pathways. We then manually reviewed the full text articles to obtain the following ITC-related annotations: (1) the names of ITC molecules in the literature; (2) the types of ITC molecules; (3) the source tissue in the communication route; (4) the target tissue in the communication route; (5) the functional descriptions of ITC molecules in the target tissue; (6) the species where ITC molecules were investigated; (7) the associated disease phenotypes; (8) the experimental methods to identify ITC molecules and their communication routes; (9) whether the ITC molecules are transported by exosomes; and (10) the PubMed ID (PMID) of the literature. In addition, because there are often multiple cell types in one tissue, determining the specific source cells and target cells of ITC molecules is also important. Therefore, if the specific source cells and target cells of ITC molecules were described in the article, the corresponding information was also extracted.

Most of the ITC molecules were classified into one of the categories of “Protein”, “RNA”, and “Metabolite”. Exosomes with unknown specific effector molecules were classified into “Exosome”. We also attempted to assign standard names to all protein and metabolite ITC molecules in order to avoid confusion. As for the source/target tissue assignments, most cases were straightforward according to the original publications, but there were also some noticeable exceptions: (a) due to the ubiquitous and dynamic distribution of immune cells, we designated the source or target tissues of molecules originating from or targeting immune cells as “Immune” [2]; (b) for the ITC molecules identified by stem cell experiments, if the source tissue of stem cells was known, we designated the source tissue as “Stem cell + Source tissue”, and, otherwise, we designated it simply as “Stem cell”; and (c) the source tissues of crosstalk mediators released by metastatic cancer cells were designated as “Primary tissue + Metastatic tissue (if any)”.

### 2.2. Proteomic and Transcriptomic Profiles and Tissue Specificity Score Calculation

A previous study has indicated that differences in mRNA and protein expression levels of some secretory protein-coding genes in tissues could potentially indicate their secretory or functioning tissues [44]. To investigate the underlying biological characteristics of ITC molecules and routes, we first introduced a tissue specificity (TS) score-based proteomic expression dataset previously generated by the GTEx team, which covers 12,621 proteins and 32 tissue types (integrated from 182 GTEx samples) [44]. The TS scores can be understood as a robust Z-score transformed expression values across tissue types, which were calculated by the R software package AdaTiSS [45]. The TS scores improved expression level comparability of the molecules across various tissues. A TS score > 0 suggested enriched expression in a specific tissue, with higher scores denoting a greater enrichment. Conversely, a TS score < 0 implied a reduced enrichment, with lower scores indicating lesser expression. We also obtained the TPM-based transcriptomic profiles (covering 36,203 genes) of the same 182 GTEx samples from the GTEx official portal and further integrated them into a TS score-based expression matrix by AdaTiSS. By procedure, comparison between protein expression abundances and RNA expression abundances was enabled. 

### 2.3. Cross-Dataset Mapping

The first issue in cross-dataset mapping is that some tissue terms were annotated at different anatomical levels. For example, some of our curated ITC entries indicate a set of tissues such as “Vessel”, “Gastrointestinal tract”, “Intestine”, and so on. On the other hand, some of the 32 GTEx tissue types are anatomically proximal and potentially functionally related (measured by inter-tissue Spearman rank correlation coefficients of proteomic and transcriptomic expression profiles, as specified in Supplementary Tables S2 and S3), such as “Heart Atrial” and “Heart Ventricle”, “Skin Unexpo” and “Skin SunExpo”, “Small Intestine” and “Colon Transverse”, and so on. Therefore, to enable the downstream analyses, we rationally constructed a mapping of tissue terms between our curated ITC data and GTEx data according to the anatomical and functional similarity of different tissues (Supplementary Table S4). For example, the term “Gastrointestinal tract” was linked with 3 GTEx tissue types including “Stomach”, “Small Intestine”, and “Colon Transverse”. Correspondingly, all the TS scores and expression abundance ranks in “Gastrointestinal tract” were calculated by averaging the corresponding indexes from these three tissues from the GTEx dataset.

In addition to the ITC proteins themselves, we also investigated the gene expression pattern of their interaction partners. The protein-protein interaction (PPI) network was constructed based on the STRING database [46], where only confident PPIs (with protein interaction scores larger than 800) were retained. We introduced gene symbol mapping to avoid confusion of gene IDs between our curated dataset and the PPI network.

### 2.4. Database Web Interface Construction

The server environment of IntiCom-DB was established under the “Linux + Apache + PHP + MySQL” framework. The database web interface was powered by HTML5 and JavaScript. The interactive table was built by using ECharts, while the interaction network visualization was enabled by d3.js plugin.

## 3. Results

### 3.1. IntiCom-DB Dataset Overview

**Figure 2:**
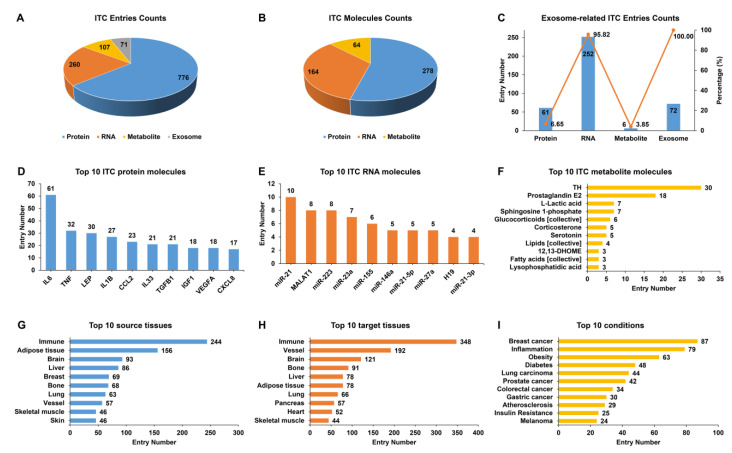
Statistical analysis of the entries in IntiCom-DB. Entry counts of (**A**) different ITC types, (**B**) different ITC molecular types, (**C**) different exosome-related ITC types, (**D**) top 10 ITC protein molecules, (**E**) top 10 ITC RNA molecules, (**F**) top 10 ITC metabolite molecules, (**G**) top 10 source tissues, (**H**) top 10 target tissues, and (**I**) top 10 physiological or pathological conditions related to ITC in IntiCom-DB.

Based on 1408 ITC entries, which were experimentally supported from 1060 publications, we established IntiCom-DB (http://rnanut.net/inticomdb), the first comprehensive database of ITC molecules and their explicit communication routes to the best of our knowledge. In the current release, IntiCom-DB covers 917 protein entries for 278 proteins, 263 RNA entries for 164 RNAs, and 156 metabolite entries for 64 metabolites, plus 72 exosome entries in which the specific molecules have not been identified (Figure 2A,B). Among both communication entries (65%) and molecules (55%), proteins account for the majority, and most of them (85%) are known secreted proteins, indicating the substantial contributions of secretory proteins to ITC [6,7,9]. The mechanism of circulating miRNAs mediated ITC has recently received considerable attention [7]. Among the 164 RNAs in IntiCom-DB, there are 137 miRNAs, accounting for more than 83%. As shown in Figure 2C, the majority of communication RNAs were delivered by exosomes, which is consistent with the previous report [18]. 

The top 10 proteins, RNAs, and metabolites with the most communication entries are shown in Figure 2D–F, respectively. IL6, miR-21, and thyroid hormone (TH) are the most studied protein, RNA, and metabolite molecules in the field of ITC, respectively. IL6 is one of the first myokines to be discovered, and its role in ITC regulating metabolism has been extensively studied [2,25]. MiR-21 is one of the most frequently upregulated miRNAs in malignant tumors and plays an important role in the development of various tumors [47]. TH is indispensable for the normal development and the regulation of metabolism, and it is closely associated with the development of metabolic syndrome and oncogenesis [48]. 

Figure 2G,H show the top 10 source tissues and target tissues with the most communication entries. The immune system provides the most ITC entries, in line with the known widely distributed immunity signaling network across various tissues [2]. ITC is altered under different physiological and pathological conditions, so we also recorded and summarized the biological condition in which each communication entry was discovered. As shown in Figure 2I, ITC is closely related to cancer, inflammation, and metabolic diseases such as diabetes and obesity. A comprehensive understanding of ITC mechanisms will contribute to the treatments against complex diseases [1,25]. Indeed, we found that a considerable fraction of communication entries was identified in research for therapeutic purposes, which were labeled with the “Therapy” condition in IntiCom-DB.

### 3.2. IntiCom-DB Web Interface

**Figure 3:**
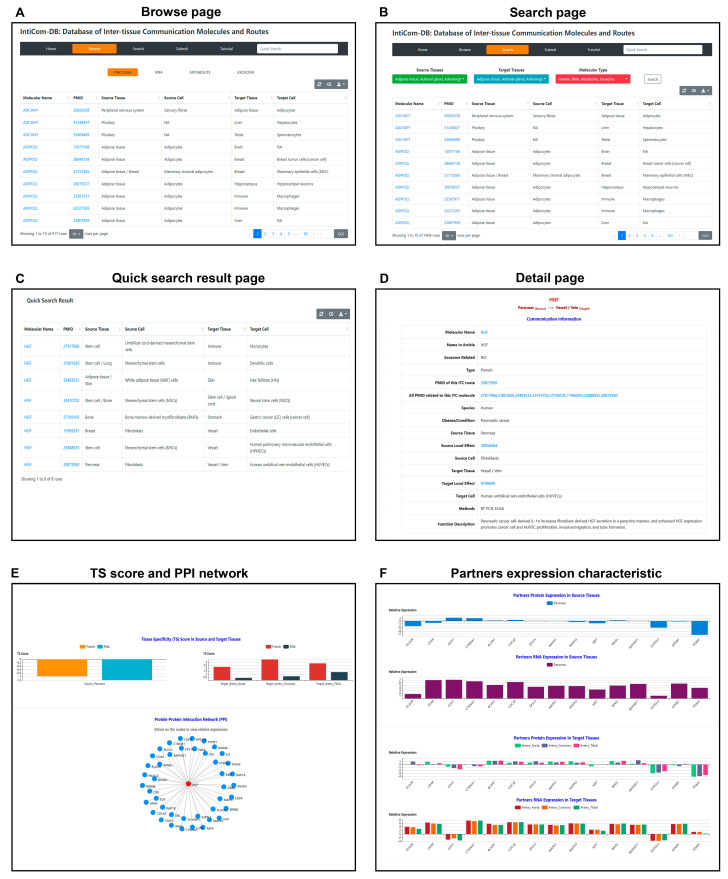
Web interface of IntiCom-DB. (**A**) Browse page: users can browse corresponding ITC entries after selecting interest molecular types. (**B**) Search page: users can search interest ITC entries by self-customized combinations of source tissues, target tissues, and molecular types. (**C**) Quick search result page: a search results specialized designed for showing the ITC entries of interested molecules, which can be accessed by inputting the molecular names (i.e., gene symbols) in the text box in navigator bar. (**D**) Detail page: basic information of ITC entries, which can be accessed by clicking the corresponding items shown in (**A**–**C**). (**E**) Tissue specificity (TS) scores and protein-protein interaction (PPI) network of ITC protein molecules. (**F**) Protein and mRNA expression abundances of ITC protein interaction partners in the source and target tissues. (**E**,**F**) The subsections of the Detail page on the database web interface.

To facilitate the community, a convenient website has been developed to browse and search all ITC data in IntiCom-DB. In the Browse page, users can browse the corresponding type of ITC entries by clicking one of the four panels: PROTEIN, RNA, METABOLITE or EXOSOME (Figure 3A). In the Search page, users can query the ITC entries of interest based on source tissues, target tissues, or molecular types in user-friendly search boxes (Figure 3B). The quick search box on the navigation bar allows users to query ITC entries based on communication molecular name, and the results will be listed on the Quick Search Result page (Figure 3C). All browsing and query results are presented as responsive tables, which contain the basic information of ITC entries, including molecular names, PMIDs of supporting publications, source tissues, and source cell types, as well as target tissues and target cell types (Figure 3A–C). The responsive tables can also be downloaded by clicking the download button in the top right corner. The molecular names can be clicked to navigate to the corresponding Detail page, where the basic information of the communication entry is shown (Figure 3D). In the Detail page, in addition to the above information, more detailed function descriptions of ITC molecules in the target tissue, and annotation for experimental methodology is shown. The molecular names of protein, miRNA, and metabolite molecules are, respectively, hyperlinked to GeneCards, https://www.genecards.org/ (accessed on 14 May 2022) [49], miRbase, https://www.mirbase.org/ (accessed on 28 Apr 2021) [50], and HMDB, https://hmdb.ca/ (accessed on 28 Apr, 2021) [51] databases for more information. In addition to miRNA, the curated RNA communication molecules also include LncRNA, circRNA, and piRNA [52], which are hyperlinked to NONCODE, http://www.noncode.org/index.php (accessed on 14 May 2022) [53], circBase, http://www.circbase.org/ (accessed on 14 May 2022) [54], and piRBase, http://bigdata.ibp.ac.cn/piRBase (accessed on 14 May 2022) [55], respectively. All PubMed IDs (PMIDs) in the database have been hyperlinked to the original PubMed articles. This will enable researchers to easily access more detailed information.

A previous study has suggested that RNA and protein expression differences of secretory proteins can provide insights into their secretory and action sites [44], and the PPI networks are also important for the analyses of ITC functions [6,56]. Therefore, expression and interaction characteristics of the ITC protein molecules, when available, have also been annotated and visualized in the Detail page for future bioinformatics-assisted xplorations of novel ITCs (Figure 3E,F). Finally, a detailed tutorial for the usage of IntiCom-DB can be found on the Tutorial page. Explorations of novel ITCs are shown in Figure 3E,F. Finally, a detailed tutorial for the usage of IntiCom-DB can be found in its Tutorial page.

### 3.3. Common Biological Characteristics of Communication Proteins Revealed by the Analysis of IntiCom-DB Data

**Figure 4:**
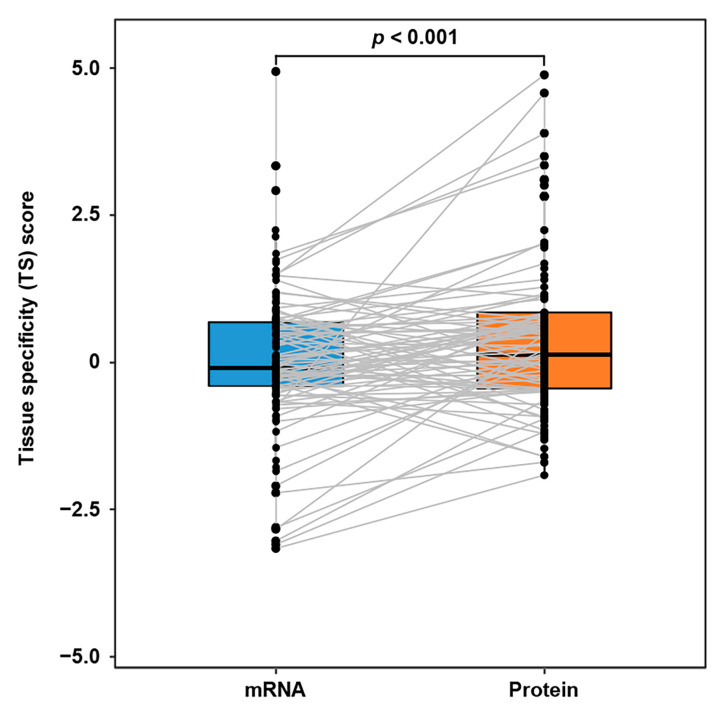
TS score difference of ITC genes at transcriptomic and proteomic levels in target tissues. The TS scores of communication proteins in the target tissues are higher than those of the corresponding mRNAs. A gray line means a mRNA-protein connection of the same ITC gene. Wilcoxon test, *n* = 146 ITC entries.

During the ITC, the communication proteins released from the source tissues are further accommodated by the target tissues. Intuitively, the abundances of communication proteins in target tissues would be relatively higher than those of the corresponding mRNAs. However, due to the lack of a sizable ITC route dataset, this assumption had not been validated with extensive cases. Here, we first introduced the tissue specificity (TS) score-based proteomic and transcriptomic profiles of GTEx dataset (covering 32 tissue types). Next, based on the IntiCom-DB data, we further extracted 146 ITC entries, where the target tissue could be linked to at least one GTEx tissue type. As expected, the Wilcoxon test has shown that the ITC molecules’ protein TS scores are indeed higher than their mRNA TS scores in target tissues (*p* = 1.13 × 10^−3^, Figure 4), which supports the hypothesis above.

Subsequently, for both the source tissues and the target tissues, we compared the protein abundances and the corresponding mRNA abundances with the random expectations. To ensure statistical robustness, we used TS score ranks across 32 tissue types but not the raw TS scores. As a result, we found in both the source tissues and the target tissues, their protein abundances are relatively higher than random expectation (Wilcoxon test, source tissue: *p* = 5.97 × 10^−3^, target tissue: *p* = 6.70 × 10^−3^). A similar tendency can also be observed for their mRNA abundances (Wilcoxon test, source tissue: *p* = 2.15 × 10^−4^, target tissue: *p* = 1.01 × 10^−2^). Furthermore, the expression pattern of the interaction partner of ITC molecules in the protein-protein interaction (PPI) network is also non-random. We observed that the interaction partners also exhibit higher abundance in both the source tissues and the target tissues than random expectation no matter what the protein level is (Wilcoxon test, source tissue: *p* = 1.50 × 10^−4^, target tissue: *p* = 3.54 × 10^−6^), or at the mRNA level, too (Wilcoxon test, source tissue: *p* = 9.50 × 10^−4^; target tissue: *p* = 2.06 × 10^−2^; Table 1). Together, these observations provide novel indictors of ITC routes from the source tissues to the target tissues, which would be helpful for prediction and exploration of novel ITC molecules and their communication routes in the future.

## 4. Discussion

ITC is a key mechanism for multicellular organisms to coordinate physiological functions, respond to environmental stress, and is closely related to many complex diseases. However, to the best of our knowledge, there is currently no dedicated database for ITC molecules and routes. 

The single cell communication databases, such as CellPhoneDB [57] and NicheNet [58], are important resources to analyze local cell-to-cell communications in one tissue. On the one hand, IntiCom-DB focuses on inter-tissue communications (ITCs) that could be distal tissue-to-tissue interactions rather than local interactions. In this sense, IntiCom-DB has good complementary features to these databases. On the other hand, though, the knowledge in IntiCom-DB may also be exploited to leverage single cell communication analysis. For example, single cell communication analysis requires the presence of mRNAs of both the receptor and the ligand in one tissue. However, for an ITC ligand, it often originates from a distal source tissue that cannot be detected by the single cell transcriptome of the target tissue. Now that we have the target tissue and interaction network annotations from IntiCom-DB, we can speculate on the presence of the ITC-related signaling if the tested tissue is the known target tissue of this ITC ligand, and the receptors (or interacting partners) of this ITC ligand are extensively up-regulated in this tissue, even without detection of the ITC ligand mRNA expression itself.

Some famous comprehensive databases such as UniProt [59] and HPA [60] cover the annotation of secretory proteins. However, known secretory proteins only constitute 47% of ITC molecules according to our data statistics. More importantly, the source and the target tissues are not annotated for many secretory proteins in these databases, since the secretory proteins are mainly annotated by either the extracellular localization observed in cultured cell lines or by computational annotation based on the presence of a signal peptide, where no tissue information could be specified. Finally, the functional description of ITC molecules is also different between the databases, as IntiCom-DB intentionally records the functional descriptions related to ITC.

Extracellular vesicles, especially exosomes, are also important mediators for intra- and inter-tissue communications. Dedicated databases, such as ExoCarta [61], Vesiclepedia [62], EVAtlas [63], and EVpedia [64], present an excellent collection of EV-related molecules by curating various high-throughput omics data that investigate the exosome compositions. However, known EV-related molecules only constitute 42% of ITC molecules according to our data statistics. Again, and more importantly, because most of these high-throughput omics assays are performed in cultured cells, most of the source tissues can, at the most, be identified for these EV components, while the target tissues and the regulatory functions therein cannot be annotated in these databases. 

Therefore, although the existing databases have included a large number of potential ITC molecules, albeit to varying degrees, they still lack key information about the source tissue, the target tissue, and the biological functions of the ITC molecules. In contrast, IntiCom-DB integrates various types of ITC molecules and accurately records detailed information, such as communication routes, from the source tissues to the target tissues, the biological functions, the disease conditions, the experimental methods, and the references for each ITC molecule through manual retrieval and extraction. This information will help to increase our understanding of the inter-tissue interaction mechanisms in complex diseases, providing clues and insights for developing new ITC-based therapeutic methods. 

Nonetheless, IntiCom-DB also has some limitations. First, compared with the database mentioned above, the amount of data recorded in IntiCom-DB is still relatively small. In fact, it is very difficult to study the functions of ITC molecules in the target tissues, which partly explains the drastic reduction of database record counts in IntiCom-DB in comparison with the abovementioned databases. When we focus on the source-to-target (instead on only focusing on the source tissue side alone), we can curate only 1408 ITC records, even with an extensive manual literature screening of 190,000 references. Second, except for some classical endocrine hormones, such as thyroid hormone, insulin, and adiponectin, which have a relative single tissue source, most protein ITC molecules are generally expressed in multiple tissues, which increases the difficulty of identifying ITC entries and routes. For example, neuropeptide Y (NPY) is a key factor regulating bone homeostasis. In addition to being expressed in non-skeletal tissues, such as the hypothalamus and adrenal glands, it is also expressed in osteoblasts, osteoclasts, and osteocytes [65]. Therefore, the role of NPY in bone may be related either to endocrine/ITC between tissues or to autocrine/paracrine effects within tissues. Due to the lack of definite evidence at the current stage, such vague ITC entries are not included in IntiCom-DB (for now). Additionally, due to the differences in tissue types between IntiCom-DB and GTEx as well as the limited number of proteins covered in the protein expression profiles based on the TS score, many protein ITC entries in IntiCom-DB were not included in the biological feature analysis due to the lack of corresponding data. In fact, among the 917 protein ITC entries, only 146 entries were included in the source tissue-related analysis. Finally, this study requires a large amount of literature curation. Although various keyword combinations and computer-assisted manual screening were employed, it is still difficult to ensure that there are no missed papers that are omitted by our pipeline.

## 5. Conclusions

IntiCom-DB, to the best of our knowledge, is the first comprehensive database of ITC molecules, containing 1408 entries of ITC information involving 278 proteins, 164 RNAs, and 64 metabolites, plus 72 exosomes with an unknown component. IntiCom-DB data also reveal common biological characteristics of ITC molecules and routes, such as TS scores of ITC molecules at the protein level, which are often higher than those at the mRNA level in target tissues, and both the ITC molecules and their interaction partners are more abundant in the source and the target tissues. We believe that IntiCom-DB will increase our understanding of human physiology and disease mechanisms, and that it will further contribute to future ITC studies.

## Figures and Tables

**Table 1:** Characterization of the protein and mRNA abundance levels of ITC genes and their interaction partners.

Molecule *	Type	Tissue *	Statistic	*p*-Value *
ITC molecules	mRNA	Target tissues	8179.5	1.01 × 10^−2^
ITC molecules	Protein	Target tissues	6290.5	6.70 × 10^−3^
ITC molecules	mRNA	Source tissues	7257.5	2.15 × 10^−4^
ITC molecules	Protein	Source tissues	5417.0	5.97 × 10^−3^
ITC molecular partners	mRNA	Target tissues	25,978.0	2.06 × 10^−2^
ITC molecular partners	Protein	Target tissues	29,329.0	3.54 × 10^−6^
ITC molecular partners	mRNA	Source tissues	21,192.5	9.50 × 10^−4^
ITC molecular partners	Protein	Source tissues	21,352.5	1.50 × 10^−4^

* Protein and mRNA abundance levels of ITC molecules and their interaction partners are higher than random expectation in source and target tissues. Wilcoxon test.

## Data Availability

The data underlying this article are freely available at http://rnanut.net/inticomdb/index.html (accessed on 31 May 2023).

## Supplementary Materials

The following supporting information can be downloaded at: https://www.mdpi.com/article/10.3390/biology12060833/s1, Table S1: PubMed retrieval keywords and literature quantity; Table S2: Inter-tissue Spearman correlation of the proteomic expression profiles in GTEx; Table S3: Inter-tissue Spearman rank correlation coefficients of transcriptomic expression profiles in GTEx; Table S4: Mapping of tissue terms between IntiCom-DB and GTEx datasets.
