# Supplementary material for: IntiCom-DB: A Manually Curated Database of Inter-Tissue Communication Molecules and Their Communication Routes
Source: Biology (Basel). 2023 Jun 8;12(6):833. doi: 10.3390/biology12060833 (PMC10294874; doi:10.3390/biology12060833)
Supplement: Supplementary file 1 [file biology-12-00833-s001.zip › biology-2383794-supplementary.pdf]

Supplemental information

IntiCom-DB: A manually curated database of inter-tissue communication molecules and their communication routes

Changxian Xiong, Yiran Zhou, Yu Han, Jingkun Yi, Huai Pang, Ruimao Zheng, Yuan Zhou

Table S1

Table S1. PubMed retrieval keywords and literature quantity.

Keywords	Literature quantity	Keywords	Literature quantity
adipose	6132	heart AND pituitary	530
adrenal AND artery	392	heart AND skin	569
adrenal AND bone	282	hormone AND axis	1755
adrenal AND heart	353	hormone AND communication	4498
adrenal AND immune	1500	hormone AND crosstalk	2109
adrenal AND kidney	275	immune AND artery	360
adrenal AND liver	342	immune AND kidney	940
adrenal AND lung	128	inter-organ-set	867
adrenal AND muscle	275	intestine	4775
adrenal AND nerve	504	kidney AND artery	502
adrenal AND pituitary	4158	kidney AND muscle	379
adrenal AND skin	280	kidney AND nerve	233
bladder	2199	kidney AND pituitary	299
bone AND artery	844	kidney AND skin	272
bone AND heart	623	liver AND artery	632
bone AND immune	1581	liver AND immune	1503
bone AND kidney	523	liver AND kidney	716
bone AND liver	531	liver AND lung	532
bone AND lung	536	liver AND muscle	563
bone AND muscle	1519	liver AND nerve	229
bone AND nerve	1048	liver AND pituitary	465
bone AND pituitary	523	liver AND skin	257
bone AND skin	688	lung AND artery	741
brain AND adrenal	4882	lung AND immune	1553
brain AND artery	2246	lung AND kidney	427
brain AND bone	1137	lung AND muscle	517
brain AND heart	1206	lung AND nerve	199

brain AND immune	2596	lung AND pituitary	268
brain AND kidney	408	lung AND skin	316
brain AND liver	628	metabolite AND axis	4004
brain AND lung	384	metabolite AND communication	6468
brain AND muscle	1533	metabolite AND crosstalk	2001
brain AND nerve	6140	multi-organ	8950
brain AND pituitary	2595	muscle AND artery	1697
brain AND skin	573	muscle AND immune	590
breast AND adrenal	140	muscle AND nerve	1636
breast AND artery	125	muscle AND pituitary	179
breast AND bone	409	muscle AND skin	642
breast AND brain	267	nerve AND artery	967
breast AND heart	210	nerve AND immune	782
breast AND immune	713	nerve AND pituitary	402
breast AND kidney	165	nerve AND skin	412
breast AND liver	245	ovary	3683
breast AND lung	713	pancreas	2869
breast AND muscle	180	peptide AND axis	3948
breast AND pituitary	227	peptide AND communication	5744
breast AND skin	301	peptide AND crosstalk	5202
colon	3790	pituitary AND artery	216
esophagus	1234	pituitary AND immune	738
exosome OR axis	769	prostate	4940
exosome OR communication	3642	salivary	2470
exosome OR crosstalk	316	skin AND artery	375
gut	4646	skin AND immune	1780
heart AND artery	3916	spleen	2460
heart AND immune	677	stomach	2585
heart AND kidney	479	testis	3153
heart AND liver	531	thyroid	4690
heart AND lung	1527	uterus	2412
heart AND muscle	4316	vagina	626

heart AND nerve	442	vein	5557

Table S2

Table S2. Inter-tissue Spearman correlation of the proteomic expression profiles in GTEx.

GTEx tissue-tissue pair	Correlation coefficient*	P-value
Artery Aorta <=> Artery Coronary	0.3832	3.63e-03
Artery Aorta <=> Artery Tibial	0.5984	< 1.00e-300
Artery Coronary <=> Artery Tibial	0.3248	1.40e-02
Brain Cerebellum <=> Brain Cortex	0.6302	< 1.00e-300
Esophagus Mucosa <=> Esophagus Muscle	-0.1164	3.77e-30
Heart Atrial <=> Heart Ventricle	0.6322	< 1.00e-300
Skin Unexpo <=> Skin SunExpo	0.6064	< 1.00e-300
Stomach <=> Small Intestine	0.3820	< 1.00e-300
Stomach <=> Colon Sigmoid	-0.2848	3.00e-194
Stomach <=> Colon Transverse	0.4071	< 1.00e-300
Small Intestine <=> Colon Sigmoid	-0.2629	2.23e-150
Small Intestine <=> Colon Transverse	0.4629	< 1.00e-300
Colon Sigmoid <=> Colon Transverse	-0.1271	1.28e-35

* Spearman rank correlation analysis of proteomic expression profiles showed that some anatomically proximal GTEx tissue types are potentially functionally related.

Table S3

Table S3. Inter-tissue Spearman rank correlation coefficients of transcriptomic expression profiles in GTEx.

GTEx tissue-tissue pair	Correlation coefficient *	P value
Artery Aorta <=> Artery Coronary	0.8189	< 1.00e-300
Artery Aorta <=> Artery Tibial	0.7596	< 1.00e-300
Artery Coronary <=> Artery Tibial	0.7347	< 1.00e-300
Brain Cerebellum <=> Brain Cortex	0.5140	< 1.00e-300
Esophagus Mucosa <=> Esophagus Muscle	0.1221	6.19e-158
Heart Atrial <=> Heart Ventricle	0.9156	< 1.00e-300
Skin Unexpo <=> Skin SunExpo	0.9284	< 1.00e-300
Stomach <=> Small Intestine	0.5464	< 1.00e-300
Stomach <=> Colon Sigmoid	0.1813	< 1.00e-300
Stomach <=> Colon Transverse	0.6824	< 1.00e-300
Small Intestine <=> Colon Sigmoid	0.2219	< 1.00e-300
Small Intestine <=> Colon Transverse	0.7236	< 1.00e-300
Colon Sigmoid <=> Colon Transverse	0.3641	< 1.00e-300

* Spearman rank correlation analysis of transcriptomic expression profiles showed that some anatomically proximal GTEx tissue types are potentially functionally related.

Table S4

Table S4. Mapping of tissue terms between IntiCom-DB and GTEx datasets.

IntiCom-DB tissue	Corresponding GTEx tissue
Adrenal gland	Adrenal Gland
Artery	Artery Aorta, Artery Tibial, Artery Coronary
Brain	Brain Cerebellum, Brain Cortex
Breast	Breast
Colon	Colon Transverse
Colon transverse	Colon Transverse
Colorectal	Colon Sigmoid
Esophagus	Esophagus Mucosa
Gastrointestinal tract	Stomach, Small Intestine, Colon Transverse
Heart	Heart Atrial, Heart Ventricle
Ileum	Small Intestine
Intestine	Small Intestine, Colon Transverse
Liver	Liver
Lung	Lung
Ovary	Ovary
Oviduct	Ovary
Pancreas	Pancreas
Peripheral nervous system	Nerve Tibial
Pituitary	Pituitary
Prostate	Prostate
Salivary gland	Minor Salivary
Pancreas	Pancreas
Skeletal muscle	Muscle Skeletal
Skin	Skin Unexpo, Skin SunExpo
Smooth muscle	Muscle Skeletal
Spleen	Spleen

Stomach	Stomach
Tendon	Muscle Skeletal
Testis	Testis
Thyroid	Thyroid
Uterus	Uterus
Vagina	Vagina
Vessel	Artery Aorta, Artery Tibial, Artery Coronary
Smooth muscle	Muscle Skeletal
Spinal cord	None
Spinal nerve root	None
Spleen	Spleen
Stem cell	None
Stomach	Stomach
Tendon	Muscle Skeletal
Testis	Testis
Thoracic DRG	None
Thyroid	Thyroid
Uterus	Uterus
Vagina	Vagina
Vein	None
Vessel	Artery Aorta, Artery Tibial
